# Preparedness to Implement Physical Activity and Rehabilitation Guidelines in Routine Primary Care Cancer Rehabilitation: Focus Group Interviews Exploring Rehabilitation Professionals’ Perceptions

**DOI:** 10.1007/s13187-020-01704-6

**Published:** 2020-02-15

**Authors:** Margit Neher, Maria Landén Ludvigsson, Anna Enblom

**Affiliations:** 1 Department of Health, Medicine and Caring Sciences, Linköping University, Linköping, Sweden; 2 Department of Rehabilitation, and Department of Health, Medicine and Caring Sciences, Linköping University, Motala, Sweden; 3 County Council of Östergötland, Linköping, Sweden

**Keywords:** Occupational therapy, Physiotherapy, Cancer survivorship, Physical activity, Primary care, Implementation

## Abstract

To explore primary care professionals’ perceptions of physical activity and other cancer rehabilitation practice in cancer survivors, investigating the preparedness to implement guidelines regarding cancer rehabilitation. We collected qualitative data through seven semi-structured focus group interviews with 48 rehabilitation professionals, with mean 9 years of experience in primary care rehabilitation (32 physiotherapists, 15 occupational therapists, and 1 rehabilitation assistant) in a primary care setting. Data was analyzed using content analysis. Primary care rehabilitation professionals expressed limited experience of cancer survivors, experienced lack of knowledge of cancer-related disability, and had doubts concerning how to treat cancer survivors. They also experienced uncertainty about where to find collaboration and support in the healthcare system outside their own rehabilitation clinic. There is a need to combine different implementation strategies to tackle multiple barriers for effective cancer survivor rehabilitation in primary care, to boost individual rehabilitation professionals’ knowledge and self-efficacy, to clarify roles and responsibilities for cancer rehabilitation across levels of care, and to develop and strengthen organizational bridges to provide adequate access to rehabilitation for cancer survivors.

## Introduction

Worldwide, about 5 million persons are diagnosed with cancer and at least 50% are expected to live at least 5 years [1]. While the incidence of cancer diseases increases worldwide, there is also a growing array of effective treatments, leading to better survival rates and growing numbers of cancer survivors [2]. Accordingly, there are increasing numbers of cancer survivors living with remaining cancer or disability related to former cancer and its treatment, for example, persistent side effects [2, 3]. The term “cancer survivor” often describes “an individual who has been diagnosed with cancer, regardless of when that diagnosis was received, who is still living” [4].

During and after the cancer treatment, medical follow-up and rehabilitation are important for quality of life [5–8]. Individualized coordinated cancer survivorship rehabilitation plans have been proposed to facilitate cancer survivors’ reintegration into daily life [2, 9], since the rehabilitation needs are unique [10]. Cancer rehabilitation provided by professionals often leads to reduced symptom burden, prevention of secondary symptoms, and reduced societal burden of care [11]. Cancer rehabilitation interventions have been seen to improve psychological and social function, work ability, and other activities of daily life [12–14]. Physical activity is suggested to have a strong impact on recurrence and survival [15]. Physical activity positively affects body composition, cardiorespiratory function, and muscle mass, and there is moderate to strong evidence that physical activity is effective for a number of cancer-related symptoms and cancer therapy–related persistent side effects, such as pain, peripheral neuropathies, fatigue, dyspnea, secondary lymphedema, altered body and self-image, and other emotional changes. The increasing numbers of observed benefits [7, 8, 16, 17] have led to guidelines regarding physical activity for cancer survivors, underscoring its importance in cancer rehabilitation [18–20]. In Sweden [20] and other countries [18, 19], guidelines recommend cancer survivors to be active in their daily lives and to practice at least 150 min of moderate intensity physical activity or 75 min of vigorous physical activity every week [18–20].

However, almost all 285,825 cancer survivors (96%) did not meet recommended physical activity guidelines 5 years after their cancer diagnosis [21], potentially due to that cancer survivors may need sufficient support from rehabilitation professionals [5, 6] to adhere to guidelines. Implementation research has clearly shown that dissemination of guidelines often does not suffice; to achieve implementation with quality, a systematic approach to implementation is recommended, starting with an investigation into stakeholder perspectives [22]. To our knowledge, no previous studies have investigated the stakeholder perspective in terms of primary rehabilitation care professionals’ preparedness for providing cancer rehabilitation. The objective of this study was to explore primary care professionals’ perceptions of physical activity and other cancer rehabilitation practice in cancer survivors, investigating the preparedness to implement guidelines regarding cancer rehabilitation.

## Methods

The study applied a qualitative study design, adopting focus group interviews for data collection [23, 24].

### Setting

The study was conducted between September 2017 and May 2018 in a primary care rehabilitation clinic located in the southeastern Sweden. After the end of cancer treatments, cancer survivors return for health issues to general practitioners and nurses at their local primary care center and to the connected primary care rehabilitation clinic. The rehabilitation clinic provides rehabilitation in four sites: one large site located in a large city and three smaller sites located in smaller towns. To coordinate and implement cancer rehabilitation in Sweden and to mitigate negative cancer-related disability, the Swedish Regional Cancer Centers in cooperation have published a national medical care program on cancer rehabilitation with a view of clinical implementation. In this document, guidelines for different aspects of cancer rehabilitation are specified (Regionala Cancercentrum, 2017).

### Inclusion of Participants

With permission from the head of the primary care rehabilitation clinic, we invited all rehabilitation professionals to participate. As part of the ordinary regular inter-professional workplace meetings, the staff was asked to share their experiences of cancer-related rehabilitation in focus group interviews. The number of the ordinary inter-professionals workplace meeting groups was seven; thus the number of focus groups was seven. The first author informed the rehabilitation professionals orally and in writing that participation was voluntary and could be ended any time and that all data would be treated and presented confidentially. Informed consent was collected.

Inclusion criteria were physiotherapists (PTs), occupational therapists (OTs), or rehabilitation assistants, working at the primary care rehabilitation clinic. No participants attending the inter-professional workplace meetings declined.

### Qualitative Focus Group Interviews

We collected qualitative interview data in the qualitative focus groups [24], including 4 to 12 participants, with duration of each session of 43–55 min. The inter-professional workplace meeting covering the focus group interview was the third in a series of four workplace meetings, focusing on facilitating of the implementation of the national medical program for cancer rehabilitation into practice. The interviewer (the first author, previously unknown to the participants) interviewed the participants using a semi-structured interview guide (Table 1). The last author (known to the participants as being responsible for facilitating the implementation of the national medical guidelines [20]) observed three of seven interviews to validate the focus of the interviews. The second author did not attend the interviews and were known to some of the participants.

**Table 1:** The semi-structured interview guide

Information, part 1: In this educational group session we would like you to explore what perceptions and thoughts you have, as rehabilitation professionals working in primary care, about the rehabilitation of late side-effects of cancer and cancer therapy, with a special focus on physical activity. This is an interactive session. That means you will learn from each other through shared reflections and discussions. I will lead the discussions while AE will assist by registering what issues we should take with us to the next session.	
Questions, part 1	
1. What different symptoms can patients present with following cancer therapy? Do you have any examples from the cases that we have previously discussed, or from your own clinical experience?	
2. Do you have experience of how these symptoms may have an impact on a person’s ability to engage in physical activity and other activities?	
3. What care can physiotherapists and occupational therapists in primary care provide for patients with different symptoms following cancer or cancer therapy?	
Information, part 2: Written information about national recommendations for physical excercise in cancer rehabilitation were handed out.	
Questions part 2	
4. How can physiotherapists and occupational therapists encourage cancer survivors to engage in physical activity? How do you collaborate in this matter?	
5. Do you have any ideas about how to improve rehabilitation practice in order to help cancer survivors to reach higher levels of physical activity?	
Information, part 3: Written information about the regional health care organization was handed out.	
Questions, part 3	
6. Do you communicate with other healthcare staff who are active in the care of these patients, but who do not belong to your own rehab organization? - What knowledge do you have about what they do when it comes to encouraging cancer survivors to be physically active? - In what way do you collaborate with other clinics involved in the rehab process?	
Information, ending the session: Now, we have talked about x, x, x and you have described y, y and y; do you have any other thoughts on today’s discussions? Thank you for sharing your experiences.	

The table presents the content of the interview guide. The information given to introduce and end the different parts of the interview is presented

^x, y^The interviewer summarized the interview in order to validate and elaborate on the recurrent topics, giving the participants the opportunity to add or clarify information

In accordance with methodological criteria for effective focus group discussions [23], the topics of the study object were discussed with enough specificity and depth to direct the discussions toward the participants’ experiences, and the interaction of different perspectives was encouraged. After a brief introduction, the semi-structured interview guide focused on the participants’ perceptions of cancer rehabilitation, of consequences of cancer and cancer therapy, and of the barriers and facilitators for cancer rehabilitation practice. The participants were also asked about their perceptions of collaborative pathways in the health care system (Table 1). At the end, the interviewer summarized the interview in order to validate and elaborate on the recurrent topics, to give the participants the opportunity to add or clarify information. The participants delivered written information regarding gender, age, profession, and number of years within primary care rehabilitation.

### Data Analysis

The audio-recorded interviews were transcribed verbatim [23]. The interviewer and the second author performed a qualitative content analysis [25] using a computer software (NVivo 11). They read the transcripts several times, evaluating patterns and content of the interview data, and marked all parts of the text that were connected to the objective of the study. The two analyzers independently classified all these relevant parts of the text (“meaning units”), into categories based on their content [25]. The analyzers focused both on quotes that were frequent and characteristic, and on quotes presenting “extra-ordinary thinking.” The categories and their accuracy were discussed until agreement was reached. The last author then independently reviewed and validated the concordance between the results from the content analysis and the content of the transcribed interviews. All authors were experienced in clinical rehabilitation (range of professional experience 18–27 years), the first author as an occupational therapist in specialized care and the second and the last author as physiotherapists in primary healthcare.

## Results

After exclusion of three individuals that did not satisfy the inclusion criteria (*n* = 2 students, *n* = 1 administrator), the study included 48 rehabilitation professionals, 40 women (83%) and 8 men (17%). Thirty-two (68%) were physiotherapists, 15 (31%) occupational therapists, and one (< 1%) was a rehabilitation assistant. The professional experience within primary care rehabilitation ranged 1–30 years, mean 9 years. Eight of the physiotherapists (25%) and 10 of the occupational therapists (67%) had at least 10 years of experience (Table 2).

**Table 2:** Characteristics of participants of the focus groups

	Focus group	No of participants	OT	Work experience in PC. mean and range (years)	PT	Work experience in PC. mean and range (years)
	1	4	3	16 (10–25)	1	0
	2	5	2	25 (20–30)	3	7 (1–17)
	3	7	2	20 (20–20)	5	9 (0–20)
	4	7	2	8 (0–16)	5	7 (1–21)
	5	12	2	14 (13–15)	10	7 (3–15)
	6	7 (of which 1 was a RA)	2	4 (0–7)	4	5 (3–8)
	7	6	2	2 (0–4)	4	5 (0–14)
Total		48	15		33	

The table presents the number (No) of participants in each focus group with different rehabilitation professions and different length of experience within primary care (PC)

*OT*, occupational therapist; *PT*, physiotherapist; *RA*, rehabilitation assistant

The qualitative data analysis revealed that participants perceived that they had little professional experience and low self-efficacy concerning rehabilitation of cancer survivors with symptoms of cancer and side effects of cancer therapy. Furthermore, they experienced uncertainty regarding available support and their own role in the healthcare system networks. Table 3 presents the categorized content of the interview data, covering three categories and nine subcategories.

**Table 3:** Categories, subcategories, and citations representing the content of the focus group interviews

Categories	Subcategories	Examples of citations
Limited practice experience	Current practice with cancer survivors	“We who are sitting here, think that we have hardly met….them, unless they have got an appointment for something else, and then it turns out that it is…..(cancer related)” (15)..
	Different or similar?	“Well, I think that they are special in that they have been through a rough (cancer) treatment…that they are impacted by that treatment in many ways… So you can take care of them the same way you as other patients, they will just have a more difficult position in their personal life…” (26).
	Red flags	“If I get a patient who has had a breast cancer and treat her for a pain in her neck, then I will treat her like any other patient…of course one always should keep the possibility of a relapse in mind…But you cannot assume that just because you have a pain in the neck region that the cancer is metastasizing” (6).
Uncertainty	Professional doubts	“If it had been another type of patient, you’d feel that you could handle it, but as soon as it’s about cancer, you get uncertain, because you have not any knowledge” (34).
	Physical advice for cancer survivors: why, who and how?	“I think it’s above all the patient’s own responsibility….and there is nothing specific about it, that one should reach this dose of exercise regardless of health or disease, just like everybody else. I think. The patients we see feel a bit worse and are in a worse state, and then it needs to be individually adapted….and then they may not be able to reach this….but that’s the advice we give to everyone…that these are recommendations for the general population” (8).
	Feelings of inadequacy	“I sometimes did not know what to do to help her (a young patient with a serious condition, author’s comment) properly…She was so severely afflicted…and it will never heal… I find that..in cases like this…one may feel a bit vulnerable” (32).
Being part of a network	Cooperation within the clinic	“Our rooms are next-door to each other, and the minute we go outside…and we have breaks together, so…it’s easy. It does not happen all the time, but it’s possible” (23)..
	Finding the way in the system	“If something is odd …..or if I would like a second opinion..an examination of a neck…something more serious..I call the doctor at the primary care unit” (9).
	Need of specialist	“The physiotherapist at the oncology department also has outpatient care, so I do not know, when they stop going there…who helps them later on…” (26). “If it’s just general physical activity advice, we rely on the (specialist unit in the) hospital” (12). “I think that there should be a group that specializes a bit in …taking care of these patients in a good way” (5). “These diseases are so diverse, and new treatments develop so fast…even the medical experts do not seem to know everything” (35).

The three categories and nine subcategories are representing the meaning units of the content of the central message of the focus group interviews. Examples of citations within each category are given. The data is presented confidentially; the code (code 1–48) represent the code number of the participant

### Limited Practice Experience

#### Current Practice with Cancer Survivors

Most participants claimed to have no experience at all being consulted by cancer survivors, whereas some of them described some limited experience. However, the cancer survivors were usually treated for something else; possible cancer-related consequences were not in focus. A seasoned physiotherapist said “*this is an unknown patient group for us, you know*” (participant 35).

#### Different or Similar?

The issue of whether or not cancer survivors should be treated differently from other patients or not was discussed. The participants pointed out that regardless of type of diagnosis, they always make an individual assessment and treat the patient accordingly. Participants with no personal experience of treating cancer survivors agreed that this would apply also for these patients.

Generally, participants did not have knowledge regarding common side effects of cancer therapy and did not ask about them, unless the cancer survivor introduced the subject. An occupational therapist with 20 years of experience said “*if the patient is there for a specific thing…we focus on that, unless the patient himself brings it up, which they rarely do…*”(9).

#### Red Flags

When reviewing new patients’ medical history, most participants asked about other diseases and health issues and some asked specifically about cancer. Some participants checked the medical files instead. Some of them talked about suspected but not yet confirmed cases of recurring cancer that they had discovered during consultations. A physiotherapist with 8 years of experience said “*Sometimes there have actually been cases where one has diagnosed that this is probably a new cancer…they have received a new…recurrence elsewhere”*.

### Uncertainty

#### Professional Doubts

The participants expressed a personal and professional uncertainty regarding what kind of rehabilitation that the cancer survivors would need. A few participants presented some experience of treating cancer-related symptoms, which were mainly related to the upper extremity, such as fractures, joint problems, and paresthesia. Lymphedema was sometimes observed as a problem, but participants did not confidently identify this as a clear-cut responsibility for primary care rehabilitation practitioners. The participants described a lack of professional knowledge about common consequences and side effects of cancer or cancer treatment and how this cancer-related disability interfere with the rehabilitation process. Their knowledge of cancer and medical regimes for cancer was described as being insufficient. “*I have no idea how anti-estrogen treatments impact the body*,” said one physiotherapist (43)*.* “*How does radiation therapy or chemotherapy influence their health?*” (44). Another aspect of uncertainty concerned the professionals’ expectations of the outcome of rehabilitation. “*How do her medications impact my treatment of her shoulder, what can I expect?*”(47). “*Maybe the medicine counteracts what we are doing, so what we do will be in vain*” (42).

Both when planning the rehabilitation and during the ongoing rehabilitation process, participants also experienced professional doubts. What is the correct treatment for these patients compared with other patients with similar symptoms? Several participants wondered about the strength of muscles and tendons “*Treatments develop so fast, and there may be a risk of side-effects that maybe…influence the structure of the body*” (35).

#### Physical Advice for Cancer Survivors: Why, Who, and How?

Although participants stated being very much aware of the beneficial effects of physical activity in many other patient groups and in the population in general, the participants expressed a lack of knowledge about how these principles should be applied to cancer survivors. A physiotherapist claimed “*If they have restrictions, they should have got them from the (oncology) clinic, but I think that normally, it isn’t that way*” (9). After reading the physical activity guidelines of the national medical care program on cancer rehabilitation [20], during the focus group session, an experienced PT said “*I am mostly surprised that there is no difference for these patients compared to other guidelines for other patients*” (27). And a colleague said “*I had never understood how very important this is*” (43).

Some expressed doubts about the applicability of the general principles of physical activity to cancer survivors and asked why cancer survivors should prioritize physical activity in their daily life. One health professional said “*I wouldn’t really be able to say how physically active I think these patients should be compared to others…*”(47)*.* Others professed to see mostly advantages of physical activity for cancer survivors, but they expressed uncertainty regarding dosage and intensity.

#### Feelings of Inadequacy

Participants discussed feelings of personal inadequacy and helplessness. One physiotherapist who is a novice in primary care but with a relatively long work experience from other areas of healthcare highlighted her concerns about the emotional aspects of meeting with a patient with a potentially harmful disease “*I’m thinking that…if you have had cancer, or have a cancer that is ongoing….that you could have a certain anxiety also…I have never met a cancer patient*”(4).

### Being Part of a Network

#### Cooperation within the Clinic

The rehabilitation professionals expressed that teamwork between PTs and OTs is not routine except in the multimodal teams aimed at pain management. However, when collaboration is needed it can be easily achieved by just talking to each other. “*Our rooms are next-door to each other, and the minute we go outside…and we take breaks together, so…it’s easy. It does not happen all the time, but it’s possible*” (23).

#### Finding the Way in the System

Participants perceived that they were not aware of any routines for reporting between the specialized clinics treating cancer and the primary care rehabilitation clinic, or the concept of contact nurse. When in need of consulting an expert, some participants had contacted the physician at the primary care unit, or another colleague that might know more *“If something is odd …..or if I would like a second opinion.., an examination of a neck…something more serious.., I call the doctor at the primary care unit*” (9).

When asked who to contact if questions arose, some participants were uncertain about where to direct the patient. For example, participants did not know which unit was responsible for treating secondary lymphedema “*Well, a long time ago, …it was at the plastic surgery clinic, but now I’m just not sure*,” said a therapist (2).

#### Need of a Specialist

Participants were not clear about the patients’ previous path in the health care system or how their need for rehabilitation were identified, and expressed varying views on how roles and responsibilities vis a vis cancer survivor rehabilitation were to be divided in the healthcare system “*The physiotherapist at the oncology department also has outpatient care, so I don’t know, when they stop going there…who helps them later on…*”(26). The responsibility of informing cancer survivors about the importance of physical activity was considered to belong to the specialist units in the hospital “*if it’s just general physical activity advice, we rely on the (specialist unit at the) hospital*” (12).

Some participants expressed the opinion that cancer issues are complex and that rehabilitation should rather be provided in other parts of the health care system than at the primary care rehabilitation clinic. One of the participants proposed the creation of a specialist team in primary care rehabilitation “*I think that there should be a group that specializes a bit in …taking care of these patients in a good way*” (5). A colleague reflected on that specialist knowledge of cancer survivor rehabilitation should be developed in other clinics “*These diseases are so diverse, and new treatments develop so fast…even the medical experts do not seem to know everything*” (35).

## Discussion

This study found that primary care rehabilitation professionals expressed limited experience of cancer survivors, experienced lack of knowledge of late cancer-related disability, and had doubts concerning how to treat the cancer survivors. They also experienced uncertainty about where to find collaboration and support in the healthcare system outside their own rehabilitation clinic.

The primary care rehabilitation professionals described a lack of clinical experience regarding cancer survivors despite their long professional experience within primary care and in spite of increasing numbers of cancer survivors. Other research points to similar deficits, but more research is needed to explore which interventions are effective for reducing the growing evidence-practice gap in cancer care [26, 27]. In a qualitative study, Birken et al. [28] found a range of individual, social, and environmental factors that influenced professional behavior. A systematic appraisal [29] found that although there is a growing number of guidelines for the use of cancer survivor plans, guideline quality is often low, providing scant information to facilitate implementation.

The rehabilitation professionals interviewed in this study experienced lack of knowledge of cancer-related disability and if cancer survivors were to be treated differently or similar to other patient groups. Participants’ main focus when asking about previous cancer disease was often to identify “red flags.” Ludvigsson and Enthoven [30] presented that physiotherapists with long work experience in primary care were indeed competent in identifying signs of serious disease that needed further investigation [30]. However, guidelines stress not only the need for surveillance of disease recurrence but also the need to pay attention to health promotion and disease prevention, monitoring, and treatment of late cancer therapy–induced side effects [2].

Participants did not feel confident in providing physical activity advice to cancer survivors, even though the guidelines are the same as for the general population and have documented effects on both health and quality of life [7, 8, 18]. Cancer survivors in general seldom adhered to physical activity guidelines without support [21]. Support and sufficient guidance has been shown to be important in providing cancer survivors with physical activity advice; a competent therapist was superior to patients accessing other sources of information without therapist guidance [31]. In a randomized controlled study (*n* = 162), cancer survivors receiving physical activity guidelines together with an exercise motivation package increased their level of physical activity. Receiving just the guidelines without no motivational support did not increase the physical activity level [32]. These studies indicate that rehabilitation professionals are valuable deliverers of physical activity guidelines, but to do this, they need to feel confident in their advice. Professionals need knowledge regarding barriers to physical activity, for example, disability and persistent side effects, and how to motivate cancer survivors to be able to offer individualized support. Since many oncology clinics do not offer any further rehabilitation after completion of cancer therapy, it is important that people with treatable disability, e.g., persistent side effects, or with other kinds of rehabilitation needs can turn to experienced and skilled rehabilitation professionals just like any other group of patients [20].

Collaboration between physiotherapists and occupational therapists within the rehabilitation clinic was perceived by participants as relatively straightforward, but the distribution of roles and responsibilities concerning cancer rehabilitation post cancer therapy was perceived as unclear: should the survivors consult the primary care or the specialized care? Both the American and Swedish guidelines for cancer rehabilitation stress the importance of coordination and collaboration across healthcare settings [10, 11, 20]. Implementation science stresses the importance of investigating barriers and facilitators at different levels and in different dimensions [33]. In this study, the inner context of the rehabilitation clinic seems to collaborate regarding cancer rehabilitation; the rehabilitation professionals did not describe external collaborations outside the clinic. Boosting the skills and knowledge of the professionals will likely not be sufficient to enable a behavior change regarding specifically collaboration. While implementation strategies targeted at individuals may be valuable; building cross-organizational bridges between levels of care is also important to clarify collaborations and division of responsibilities for cancer survivorship care [34, 35].

There are different methodology aspects of the study to be discussed. Interviewing 48 rehabilitation professionals in seven focus groups provided extensive data, and the content was assessed as rich. In the last focus group interviews, the rehabilitation professionals expressed similar experiences and perceptions like those in previous focus groups.

The multi-disciplinary research team’s variety of individual experiences in clinical rehabilitation, in clinical research, and in theoretical knowledge may have influenced the data analysis, entailing both advantages and limitations. By providing information about the participants, the interview situation, the background of the authors, and the data analysis procedure, we sought to improve study confirmability. Data quality was enhanced by the possibility to uncover the participants’ experiences and perceptions of factors that were brought up by the other focus group participants, which may be difficult to achieve with individual interviews [24]. Two authors analyzed the data, and a third author critically appraised the analysis; this triangulation [36] is strengthening the trustworthiness of our findings. Due to the qualitative design, the results will not be automatically transferable to other contexts. However, besides being as true as possible to the content of data, we compared our findings with other studies concerning implementation of cancer rehabilitation to explore the theoretical generalizability of our findings.

The study points to the need of further research concerning effective implementation of evidence-based cancer rehabilitation routines in primary care to be able to achieve the best possible health and quality of life for cancer survivors. The study concludes that primary care rehabilitation professionals expressed little experience of cancer survivors, had limited knowledge of cancer-related disability and how to treat them, and perceived uncertainty about where to find network support in the healthcare system. These findings point to the need to combine different implementation strategies to boost individual rehabilitation professionals’ professional knowledge and self-efficacy, to clarify roles and responsibilities for cancer rehabilitation across levels of care, and to develop and strengthen organizational bridges to provide adequate access to rehabilitation for cancer survivors.

## Data Availability

The authors have full control of all primary data and agree to allow the journal to review all data if requested.
